# Study on Volatile Chemicals as Spoilage Indexes of Salmon by HS-SPME-GC-MS Technique during Non-Frozen Storage

**DOI:** 10.3390/molecules28010013

**Published:** 2022-12-20

**Authors:** Guanhua Xuan, Miaorong Guo, Hong Lin, Jianxin Sui, Jingxue Wang

**Affiliations:** Food Safety Laboratory, College of Food Science and Engineering, Ocean University of China, Qingdao 266003, China

**Keywords:** solid-phase microextraction, gas chromatography-mass spectrometry, salmon, freshness, spoilage indexes

## Abstract

Freshness is the most fundamental and important factor to assess raw fish quality. The purpose of our study was to determine the potential spoilage indexes of salmon during non-frozen storage by using headspace solid-phase microextraction (HS-SPME) followed by gas chromatography-mass spectrometry (GC-MS). More than 300 volatile compounds in salmon were detected when sensory scores declined gradually following the quality changes of salmon at different temperatures. And there were 27 and 31 compounds that showed concentration variations when stored at 4 °C and 25 °C, respectively. Among them, the contents of 1,3-di-tert-butylbenzene, acetic acid, and 3-methyl-1-butanol increased significantly in the later storage period and were in accordance with the salmon’s qualities. The present study provides valuable information on the volatile chemical spoilage indexes that are closely related to the freshness of salmon, which may provide an efficient alternative way for quality evaluation of salmon.

## 1. Introduction

The quality of aquatic products is defined by several criteria, such as food safety, origin, traceability of the products, nutritional quality, and freshness [1,2]. Among them, the freshness of products attracted more attention, especially raw food, which is highly perishable [3,4]. Raw salmon (*Oncorhynchus* spp.) is often used for sashimi because of its delicate meat, smooth taste, bright colors, and rich nutrients [5]. However, the shelf-life of salmon is very short, which is mainly due to the existence of a series of damage mechanisms, such as autolytic degradation, microbial spoilage, and lipid oxidation [6]. There is an urgent need to develop analytical methods, which can be easily operated and accurately evaluate fish freshness.

Sensory evaluation, microbiological, and chemical methods are used routinely to evaluate fish freshness [7]. Sensory attributes mainly include appearance, texture, odor, and color [8]. Sensory evaluation is the most used method to assess freshness, however, it is highly dependent on the consumer’s level of perception and knowledge of the product [9]. The specific spoilage organism (SSO) is the main cause that leads to spoilage [10], and thus spoilage bacteria could be used as a key indicator of fish freshness. But its main disadvantage is that the procedure to determine bacterial numbers is quite time consuming. For chemical indicators, the total amount of volatile bases (TVB-N) has been extensively used as fish freshness indicators. Unfortunately, this freshness index sometimes cannot fully reflect the real quality changes of fish accurately, and it is not applicable to all fish species [2,11]. As an example, the T-VBN value is more suitable as a spoilage index for the fish whose specific spoilage organisms are *Shewanella* spp., but not for *Pseudomonas,* while various studies have shown that the specific spoilage organism in salmon includes *Pseudomonas* [12]. Hence, the development of spoilage indexes that can accurately evaluate fish freshness is still a challenge.

The volatile organic compounds (VOCs) can be produced by the decomposition of available nutrients and the metabolic pathway of spoilage microbiota [13]. It has been found to change significantly with the extension of storage time and the growth of microorganisms [14,15]. Miyasaki [16] analyzed the difference in flavor changing among several fishes popular as “sashimi” and proposed that VOCs, such as 1-heptanol, (E)-2-octenal, (E)-2-hexenal, 1-pentanol, (E,E)-2,4-heptadienal, 2,4-hexadienal, 1-hexanol, 4-heptenal, were thought to be appropriate markers for monitoring the freshness of “fresh” fish except for white meat fish. Anagnostopoulos [17] revealed that 2-methylbutanal, 3-methylbutanal, 3-methyl-1-butanol, ethanol, 2,4 octadiene (two isomers), ethyl lactate, acetaldehyde, and (E)-2-penten-1-ol could be used as potential spoilage markers of red seabream. Although the specific VOCs differ in different fish species, these findings indicate a potential role of the VOCs as freshness or spoilage markers of seafood.

Chromatography and mass spectroscopy are common techniques for fish freshness assessment and quality evaluation [18]. Solid-phase microextraction (SPME) is a new method of sample treatment without solvent, which integrates sampling, extraction, concentration, and sample injection into one [19,20,21]. Here, the combination of HS-SPME and GC-MS was used to accurately determine and quantify the volatile compounds, especially for those with the lower threshold value in stored salmon. The main objective of this study is to determine the potential spoilage indexes that could accurately reflect salmon quality, which provides a reference for the determination of salmon freshness.

## 2. Results

### 2.1. Sensory Analysis

**Figure 1:**
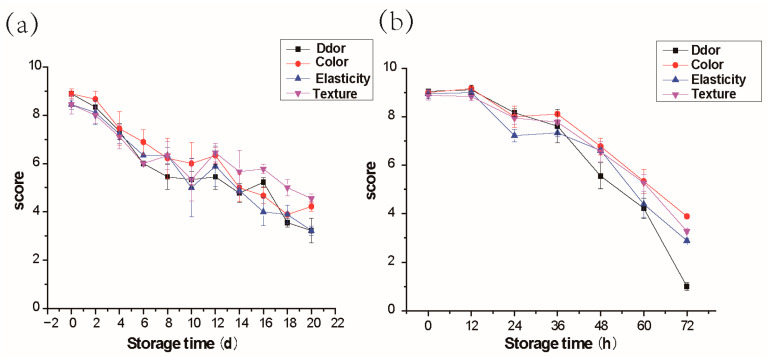
Changes of sensory in salmon stored at 4 °C (**a**) and 25 °C (**b**). Error bars are representative of mean ± standard error.

The sensory changes of salmon during storage at 4 °C and 25 °C are shown in Figure 1. The odor, color, elasticity, and texture decreased during salmon storage. Scores of four parameters were less than 6 points in 6 days, suggesting the shelf time of salmon at 4 °C is 6 days, and the shelf time of salmon at 25 °C is 48 h. The texture score decreased significantly in the early stage, and the elasticity and odor scores decreased rapidly in the later period when stored at 4 °C. For comparison, the elasticity score decreased significantly in the early stage of storage at 25 °C, and the odor score decreased rapidly in the later stage. Overall, the odor changes were more significant especially in the storage at 25 °C.

### 2.2. Principal Component Analysis

**Figure 2:**
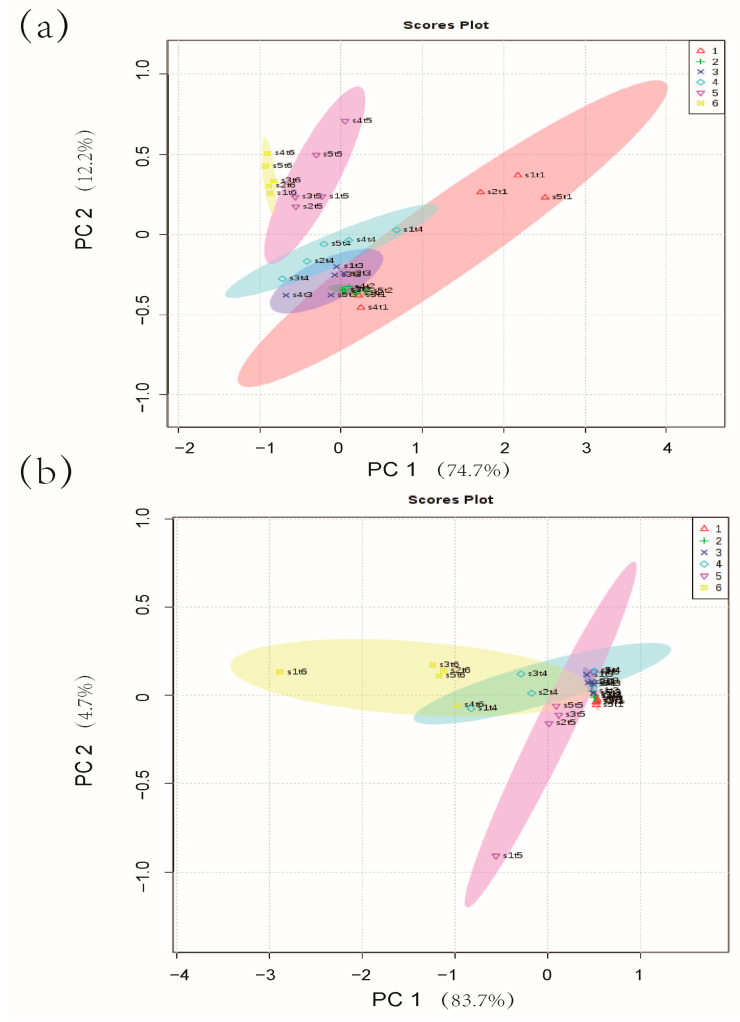
PCA analysis of compounds. (**a**) Storage time at 4 °C: class 1 represents 0 day, class 2 represents 4 day, class 3 represents 8 day, class 4 represents 12 day, class 5 represents 18 day, and class 6 represents 20 day; (**b**) Storage time at 25 °C: class 1 represents 0 h, class 2 represents 24 h, class 3 represents 36 h, class 4 represents 48 h, class 5 represents 60 h, and class 6 represents 72 h.

Principal component analysis (PCA) can derive the most important and contributory factors from the multivariate variables, in order to observe and compare the spatial distribution differences of principal component analysis values of different samples [22]. PCA was performed on the results of different storage temperatures, and the sum of the first principal component and the second principal component was greater than 85%, which can be used to represent most of the compounds. Most of the sample groups in the 4 °C storage process, except for the 0th day group and the 4th group, can be better separated based on storage time, indicating that the salmon samples have changed significantly with the prolonged storage time (Figure 2a). The first two groups (days 0 and 4) intersected each other and could not be distinguished, indicating that the samples did not change significantly within 0–4 days of storage time. At the same time, the samples in the group on the 0th day were far apart, indicating that the errors between the parallel samples of the group were large. In Figure 2b, there is a significant cross-coincidence between the sample groups at different time points during storage at 25 °C, which is difficult to completely distinguish, especially in the 0th h group, the 24th h group, and the 36th h group. Full overlap indicates that the sample change was not obvious within the first 36 h of storage at 25 °C; however, the differences between the 48th h group, the 60th h group, and the 72nd h group were significant, indicating that the rapid change in samples occurred in the later storage period.

### 2.3. The Change of Volatile Organic Compounds (Non-Targeted Detection)

**Figure 3:**
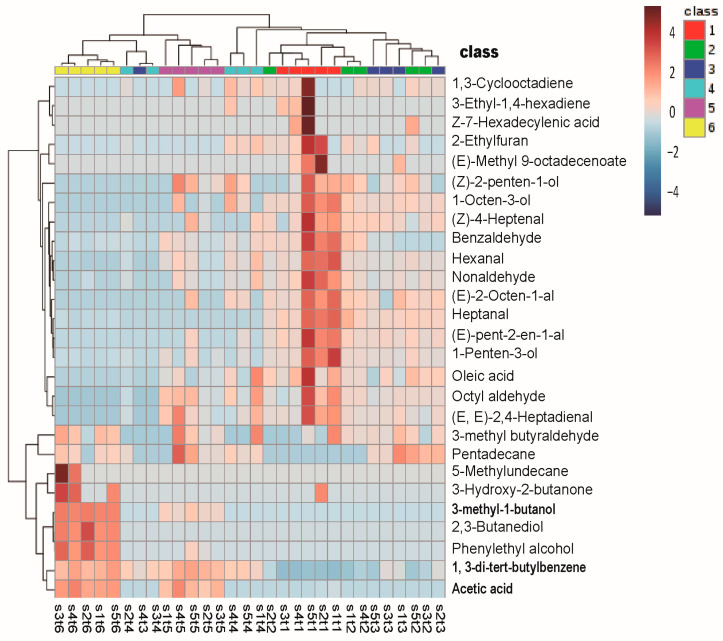
Clustering result shown as heatmap. Storage time at 4 °C: class 1 represents 0 day, class 2 represents 4 day, class 3 represents 8 day, class 4 represents 12 day, class 5 represents 18 day, and class 6 represents 20 day. Each experimental group is set with five parallels. The x-axis is expressed in the form of SaTb, where “a” represents the parallel value and “b” represents the class of different storage days. The most and least intense relative abundance values are depicted in red and blue, respectively.

**Figure 4:**
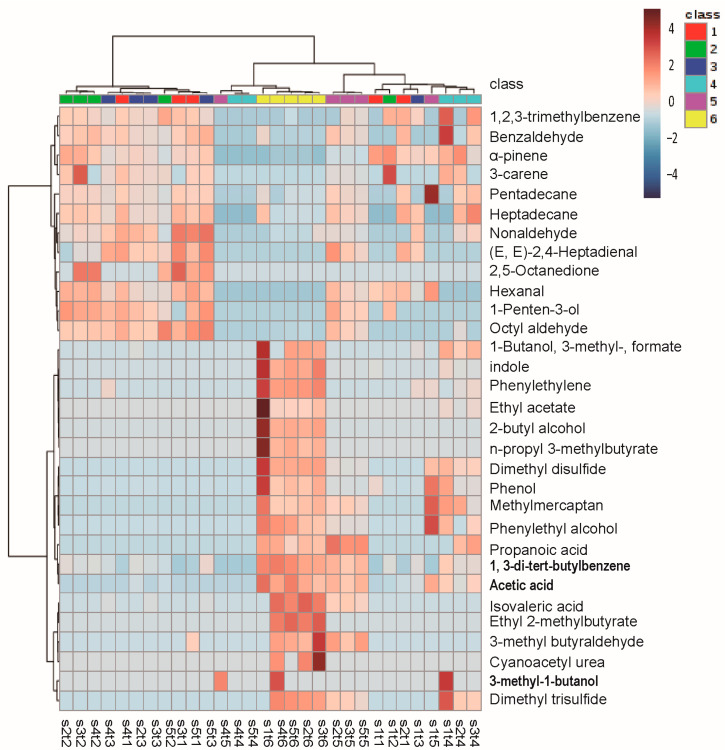
Clustering result shown as heatmap. Storage time at 25 °C: class 1 represents 0 h, class 2 represents 24 h, class 3 represents 36 h, class 4 represents 48 h, class 5 represents 60 h, and class 6 represents 72 h. Each experimental group is set with five parallels. The x-axis is expressed in the form of SaTb, where “a” represents the parallel value and “b” represents the class of different storage days. The most and least intense relative abundance values are depicted in red and blue, respectively.

By using the HS-SPME-GC-MS method, more than 300 compounds were detected by SCAN mode in salmon during storage at 4 °C and 25 °C. A significant increase in compound species and peak area were found in the spoiled salmon compared with the fresh samples. In addition, the species and peak area of the compounds in the spoiled salmon at 25 °C were all more than (*p* < 0.05) spoiled salmon stored at 4 °C (Supplementary Materials: Figure S1). After initial screening, 27 and 31 compounds with significant changes at 4 °C and 25 °C, respectively, were subjected to further cluster analysis (Tables S2 and S3). Results of PCA-based cluster analysis showed that 27 compounds (change at 4 °C) were divided into two clusters. On the first hierarchical clustering, 18 compounds with high contents were found in fresh samples, and their concentration decreased as storage time increased and spoilage was initiated. On the second hierarchical clustering, the concentration of 7 components, that is 5-methyl-undecane, 3-hydroxy-2-butanol, 2,3-butanediol, phenylethyl alcohol, 3-methyl-1-butanol, 1,3-di-tert-butylbenzene, and acetic acid increased with the deepening of corruption (Figure 3). When stored at 25 °C, a total of 19 compounds in the second hierarchical clustering also showed an increasing trend in concentration during storage (Figure 4).

After comparative analysis, we identified 11 common compounds in the above 27 and 31 compounds with significant changes at 4 °C and 25 °C, respectively. It includes 3-methyl-1-butanol, 1,3-di-tert-butylbenzene, acetic acid, phenylethyl alcohol, and 3-methyl-butanal, with their contents increased during storage. While the contents of nonanal, hexanal, 1-penten-3-ol, octanal, (E,E)-2,4-heptadienal, and benzaldehyde were significantly reduced during spoilage. We noticed that the tendency and content of 3-methyl-1-butanol, 1,3-di-tert-butylbenzene, and acetic acid significantly changed over time, which may be used as the potential indexes for salmon freshness assessment (Figure 3 and Figure 4).

### 2.4. Quantitative Verification of Salmon Spoilage Indexes (Targeted Detection)

**Figure 5:**
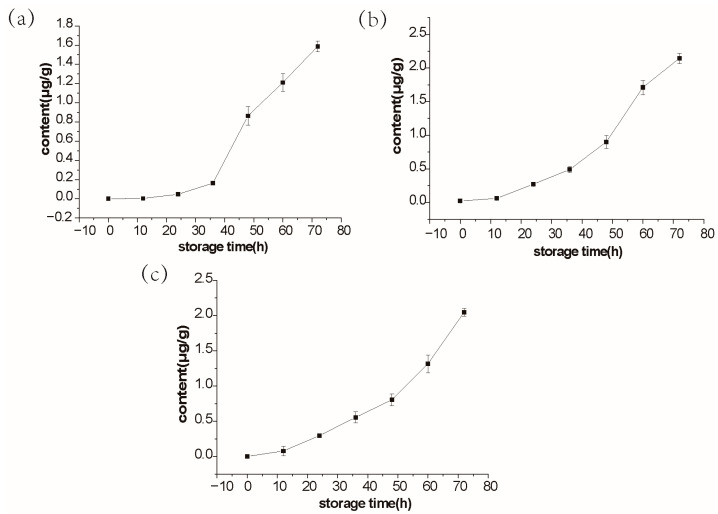
Content changes of three compounds of salmon at 25 °C as measured by GC-MS: (**a**) 3-methyl-1-butanol; (**b**) 1,3-di-tert-butylbenzene; (**c**) acetic acid.

The standard curves of 3-methyl-1-butanol, 1,3-di-tert-butylbenzene, and acetic acid were established, with the correlation coefficients (R^2^) all greater than 0.99 (Figure S2). Further study on their variation trends in salmon during 25 °C storage showed that 3-methyl-1-butanol, 1,3-di-tert-butylbenzene, and acetic acid displayed an increasing trend with the maximum values, respectively, 1.59 μg/g, 2.14 μg/g, and 2.05 μg/g during storage with the extension of time (Figure 5), which verified the previous results of non-target experiments (Tables S2 and S3).

Using KEGG (Kyoto Encyclopedia of Genes and Genomes) codes of endogenous metabolites, a pathway analysis by the MetaboAnalyst tool [23] was carried out. We performed pathway analysis on the above three spoilage indexes, only acetic acid has a related metabolic pathway in KEGG. Acetic acid is involved in phosphonate and phosphinate metabolism, sulfur metabolism, selenoamino acid metabolism, pyruvate metabolism, and glycolysis or gluconeogenesis (Figure S3). Moreover, the metabolic pathways in which salmon change significantly during storage are phosphonate and phosphinate metabolism, sulfur metabolism. As shown in Figure S3a,c, the main sources of acetic acid are phosphonoacetate, allocystathionine, and sulfate.

## 3. Discussion

This study focused on the correlation between volatile compounds and the freshness of fish. In general, a significant decline was observed in the sensory properties during storage. The results of sensory evaluation revealed that out of three quality descriptors tested (odor, color, and texture), odor as the key parameter for salmon acceptance, showed the sharpest decrease in sensory scores (Figure 1). Using the HS-SPME-GC-MS method, we determined more than 300 kinds of volatile compounds during the salmon storage, and the level of 3-methyl-1-butanol, 1,3-di-tert-butylbenzene, and acetic acid changed with fish freshness. Further quantification of 3-methyl-1-butanol, 1,3-di-tert-butylbenzene, and acetic acid, the concentrations were respectively 1.59 μg/g, 2.14 μg/g, and 2.05 μg/g (Figure 5). Since the three volatile compounds are closely related to the freshness of salmon, which could be used as a reliable indicator of fish freshness.

Volatile compounds have been shown to change during storage, which can be used as good indicators of fish spoilage or shelf-life instead of sensory evaluation [24]. Our PCA results indicated that salmon has a slow metamorphism rate in the early stage of storage at 25 °C, and the rate of decay in the later stage is accelerated (Figure 2). We monitored the variation of volatile compounds in salmon and found 3-methyl-1-butanol, 1,3-di-tert-butylbenzene, and acetic acid contents correlated with sensory results (Figure 5). 3-methyl-1-butanol and acetic acid have been reported to be important fish freshness indicators. The content of 3-methyl-1-butanol increased with storage time and correlated with the T-VBN and sensory results of traditional fish freshness indicators [25,26,27]. 3-methyl-1-butanol was identified as a potential spoilage index closely related to salmon freshness [12,28]. Acetic acid was also found to be associated with salmon spoilage [29]. Macé et al. [30] verified that acetic acid was the source of the acid odor of spoiled salmon. Our research was consistent with the previous finding that 3-methyl-1-butanol and acetic acid are correlated with fish freshness. Differently, we investigated the volatile substances of salmon under non-refrigerated conditions (4 °C and 25 °C) more comprehensively based on the targeted and non-targeted detection, which could reflect the quality of salmon more accurately combined with the sensory analysis. In addition, 1,3-di-tert-butylbenzene is a newly identified fish freshness indicator, with its contents changed obviously with the extension of the storage period, especially in the early stage of storage at 4 °C (Table S2).

Most of the volatile compounds identified in this study, such as alcohols, aldehydes, sulfide, and esters, have been reported as metabolites of microbial origin [12,31]. The production of 3-methyl-1-butanol was usually attributed to *Pseudomonas* spp. [32]. *B. thermosphacta, S. putrefaciens, P. phosphoreum,* and lactic acid bacteria, were linked with the synthesis of acetic acid [30,33]. However, the microorganism contribution and chemical pathways leading to the formation of 1,3-di-tert-butylbenzene in salmon are not clear, which requires further investigation and verification.

## 4. Materials and Methods

### 4.1. Materials and Reagents

Fresh salmons (*Oncorhynchus* spp.) (weight 8–10 kg, length 110–130 cm) were provided by Oriental Marine Technology Co. Ltd. of Shandong Province (Yantai, China). The time between fish caught by the fisherman and the analysis was within a day; throughout this period the fish were stored on ice. The fish were filleted and immediately sampled on arrival at our laboratory. The salmon samples weighing 25 g were placed in sterile polyethylene stomacher bags individually and were stored under different conditions for further analysis. Each index of each fillet was measured only once.

2,4,6-trimethylpyridine (≥99%), sodium chloride, 3-methyl-1-butanol (≥99.6%), 1,3-di-tert-butylbenzene (≥95%), and acetic acid (≥99.9%) were purchased from Chinese medicine Group Chemical Reagent Co., Ltd. (Shanghai, China).

### 4.2. Sensory Evaluation

Salmons were placed at 4 °C for 0, 2, 4, 6, 8, 10, 12, 14, 16, 18, and 20 days, and placed at 25 °C for 0, 12, 24, 36, 48, 60, 72 h for sensory evaluation. According to the method previously reported [34], the four quality parameters change, namely odor, color, elasticity, and texture were evaluated by six trained sensory panelists. Overall acceptability was calculated by taking the average of all four parameters. Six trained panelists from the laboratory staff were requested to assess the salmon fillets independently. The evaluation criteria is shown in Table S1.

### 4.3. HS-SPME-GC-MS Analysis

HS-SPME extraction

The volatile compounds were extracted according to the method described in Hua Feng [35] with minor modifications. In brief, approximately 2 g minced fish, 4 mL saturated sodium chloride solution, and 5 μL 2, 4, 6-trimethylpyridine solution (Internal Standard) were added to the headspace bottle. The 50/30 μm DVB/CAR/PDMS fiber was applied to extract the volatile organic compounds from the headspace over salmon samples. Each vial was equilibrated at 50 °C for 30 min, followed by the volatile organic compounds extracting for 30 min, unless otherwise stated. The volatile organic compounds were then thermally desorbed in the SPLITLESS mode into the injector port heated to 260 °C for 5 min using a narrow inlet SPME liner.

SPME-GC-MS analysis

GC-MS analysis of extracted volatiles was performed using GC (6890N series Agilent Technologies, Palo Alto, CA, USA) coupled to MS (Agilent 5973) equipped with a fused silica capillary column HP-INNOWax (30 m × 0.25 mm × 0.25 μm). The injector temperature was set at 260 °C to allow the thermal desorption of volatile organic compounds. The carrier gas was high-purity helium with a flow rate of 1 mL/min. The following GC oven temperature program was applied: 40 °C (for 5 min) → ∆T 5 °C/min → 250 °C (for 5 min). The GC-MS interface temperature was 260°C, and helium was used as a carrier gas with a column flow rate of 1.0 mL/min. The peaks of the separated volatile organic compounds were analyzed with a quadrupole mass spectrometer working in electron ionization (EI) mode at 70 eV at 230 °C. The mass spectra were scanned in the range of 33–489 *m*/*z*.

Data and bioinformatics analysis

The peak areas were acquired using SCAN (non-targeted detection) and SIM (targeted detection) by FASST mode. The preliminary identification of individual volatile organic compounds was based on the comparison of their mass spectra with the NIST database (https://www.nist.gov/srd, accessed on 14 March 2019) using a similarity index (SI > 70%) and comparison of retention times with available analytical standards. MetaboAnalyst 4.0 (https://www.metaboanalyst.ca/faces/home.xhtml, accessed on 9 February 2019) online software was used for data analysis and plotting.

### 4.4. Statistical Analysis

The experiment data described in the sensory analysis were expressed as the means ± SD. All the data of quality traits were analyzed with the use of SPSS statistical software, version 25.0 (IBM, Armonk, NY, USA). *p* < 0.05 was regarded as a significant difference.

## 5. Conclusions

We examined the sensory changes of salmon during storage at 4 °C and 25 °C. The sensory scores decreased gradually during storage. Salmon has a slow metamorphism rate in the early stage, and the rate of decay in the later stage is accelerated. More than 300 volatile compounds in salmon were detected by using the HS-SPME-GC-MS technique when sensory scores declined. Among them, 3-methyl-1-butanol, 1,3-di-tert-butylbenzene, and acetic acid contents significantly varied from fresh to different decay stages and were selected for quantitative determination. The results indicate that the three compounds are closely related to the freshness of salmon and are suitable for indicating the quality of salmon as potential spoilage indexes.

## Data Availability

The data supporting the findings of this study are included in this article.

## Supplementary Materials

The following supporting information can be downloaded at: https://www.mdpi.com/article/10.3390/molecules28010013/s1, Figure S1: GC-MS chromatogram of Salmon. (a) day 0 during 4 °C storage; (b) day 20 during 4 °C storage; (c) 0 h during 25 °C storage; (d) 72 h during 25 °C storage; Figure S2: The standard curves of (a) 3-methyl-1-butanol, (b) 1,3-di-tert-butylbenzene, and (c) acetic acid were established, with the correlation coefficients (R^2^) all greater than 0.99; Figure S3: Pathway analysis of acetic acid by MetaboAnalyst 4.0. (b) Each node represents one metabolic pathway, arranged by p-value (from pathway enrichment analysis) on Y-axis and pathway impact value (from pathway topology analysis) on X-axis. Nodes are composed of two factors: node color based on p-value with a chromatic scale from white (highest p-value) to red (lowest p-value) and node radius determined based on pathway impact value. (a), (c), (d), (e), and (f) are partial information on acetic acid in each metabolic pathway; Table S1: Sensory evaluation criteria of salmon; Table S2: Relative content changes of 27 volatile compounds of salmon during storage at 4 °C; Table S3: Relative content changes of 31 volatile compounds of salmon during storage at 25°C; Table S4: The characteristic ions and retention times of 3-methyl-1-butanol, 1,3-di-tert-butylbenzene, Acetic acid under SIM mode.
