# Supplementary material for: Study on Volatile Chemicals as Spoilage Indexes of Salmon by HS-SPME-GC-MS Technique during Non-Frozen Storage
Source: Molecules. 2022 Dec 20;28(1):13. doi: 10.3390/molecules28010013 (PMC9822355; doi:10.3390/molecules28010013)
Supplement: Supplementary file 1 [file molecules-28-00013-s001.zip › molecules-2062076-supplementary.pdf]

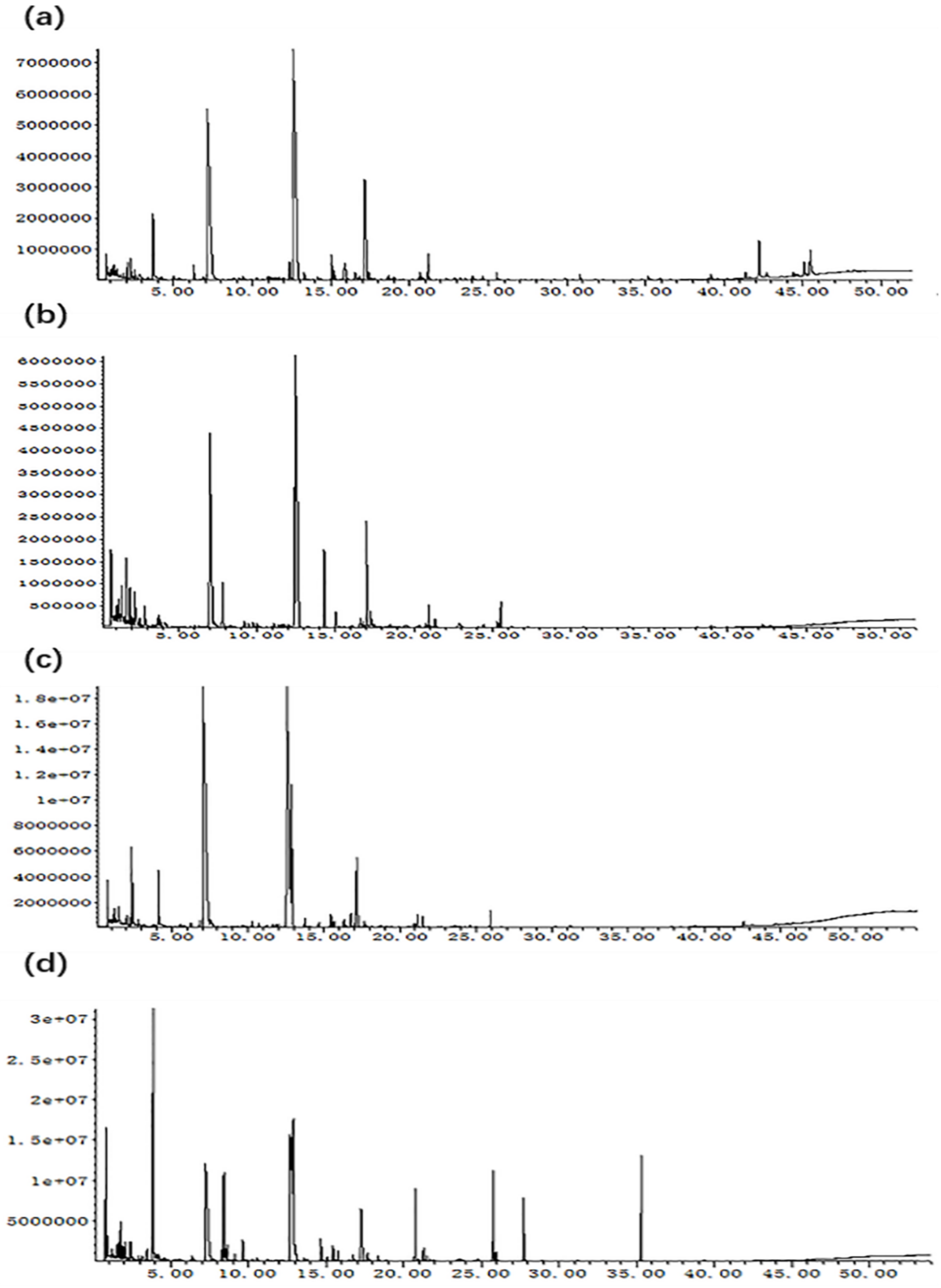

Figure S1. GC-MS chromatogram of Salmon. (a) day 0 during 4 °C storage; (b) day 20 during 4 °C storage; (c) 0 h during 25 °C storage; (d) 72 h during 25 °C storage.

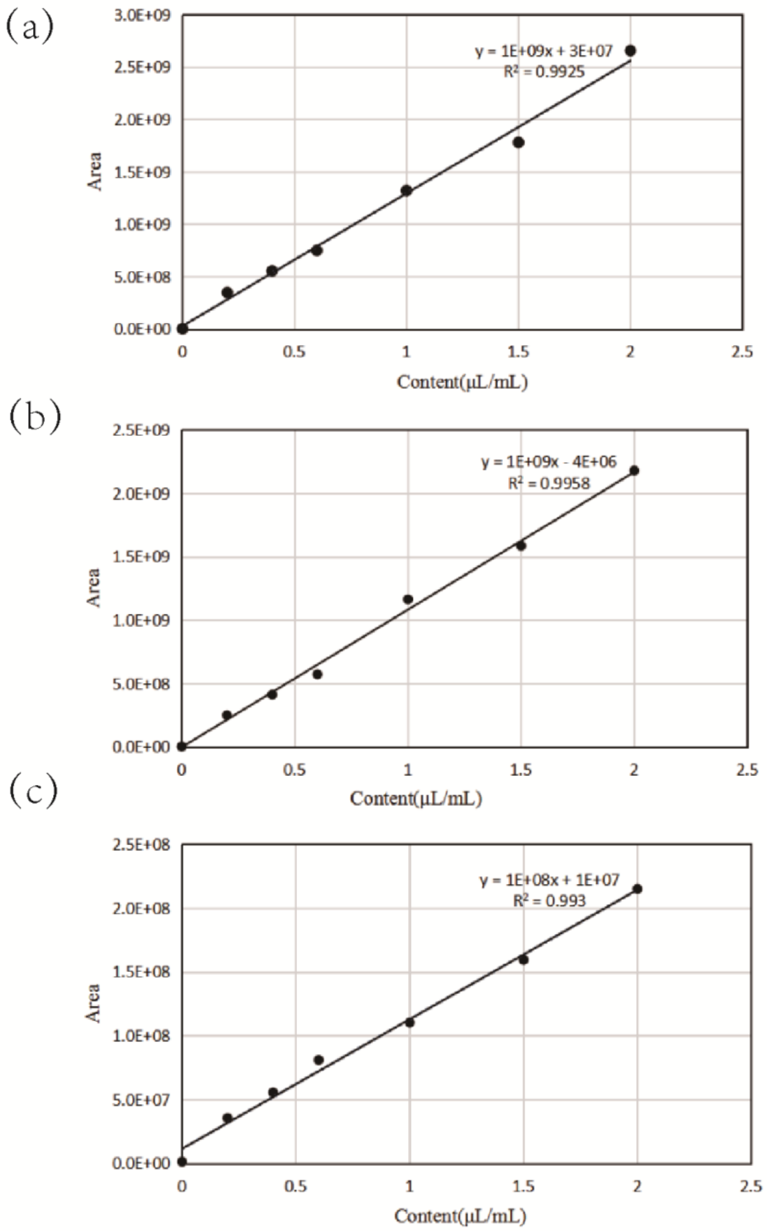

Figure S2. The standard curves of (a) 3-methyl-1-butanol, (b) 1,3-di-tert-butylbenzene, and (c) acetic acid were established, with the correlation coefficients (R2) all greater than 0.99.

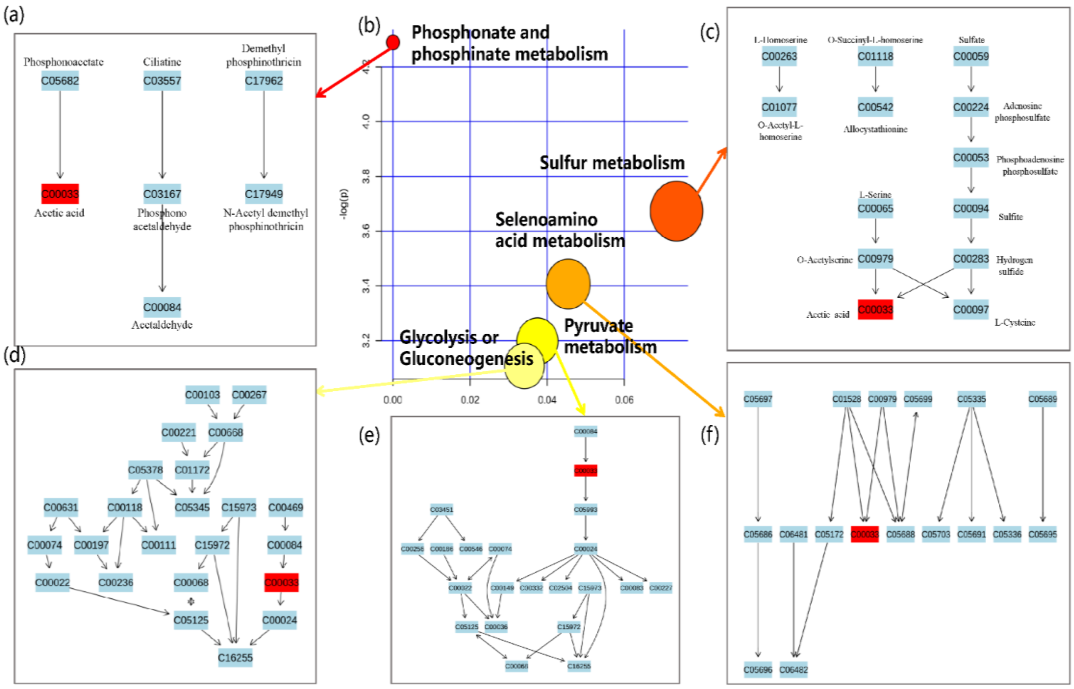

Figure S3. Pathway analysis of acetic acid by MetaboAnalyst 4.0. (b) Each node represents one metabolic pathway, arranged by p-value (from pathway enrichment analysis) on Y-axis and pathway impact value (from pathway topology analysis) on X-axis. Nodes are composed of two factors: node color based on p-value with a chromatic scale from white (highest p-value) to red (lowest p-value) and node radius determined based on pathway impact value. (a), (c), (d), (e), and (f) are partial information on acetic acid in each metabolic pathway.

Table S1. Sensory Evaluation Criteria of Salmon.

Score	Odor	Color	Elasticity	Texture
8-10	Fresh seaweedy, neutral	Bright, iridescent pigmentation	Fish is elastic.	In rigor
6-8	Cucumber, metal	Pigmentation bright but not lustrous	Less elastic, finger mark disappears rapidly	The muscle tissue is less tight
4-6	Sour	Pigmentation in the process of becoming discoloured and dull	Poor elasticity, finger leaves mark over 3 seconds	The muscle tissues is loose
2-4	Rotten	Yellowish	No elastic.	The muscle fiber is blurry

Table S2. Relative content changes of 27 volatile compounds of salmon during storage at 4 °C.

Compounds	Relative content（μg/g）									
	Day 0	Day 2	Day 4	Day 6	Day 8	Day 10	Day 12	Day 14	Day 18	Day 20
2-Ethylfuran	0.33	0.05	0.08	0.01	0.04	0	0	0	0	0
Hexanal	5.19	2.12	1.79	2	1.55	2.22	2.05	1.71	1.51	0.07
(E)-pent-2-en-1-al	0.45	0.14	0.16	0.11	0.16	0.21	0.03	0.05	0.04	0
1-Penten-3-ol	1.19	0.36	0.36	0.30	0.28	0.35	0.09	0.11	0.18	0.03
Heptanal	0.31	0.14	0.15	0.12	0.12	0.12	0	0.01	0	0.01
(Z)-4-Heptenal	0.15	0.05	0.07	0.07	0.08	0.07	0.03	0.03	0.03	0
Octyl aldehyde	0.21	0.13	0.12	0.15	0.10	0.17	0.13	0.0	0.04	0
(Z)-2-penten-1-ol	0.19	0.09	0.12	0.05	0.05	0.08	0.09	0.06	0.04	0
Nonaldehyde	0.44	0.18	0.15	0.12	0.12	0.10	0.15	0.04	0.08	0.01

(E)-2-Octen-1-al	0.16	0.05	0.08	0.08	0.07	0.10	0.04	0.10	0.02	0
1-Octen-3-ol	1.29	0.49	0.62	0.60	0.57	0.57	0.42	0.42	0.26	0
(E, E)-2,4-Heptadienal	0.79	0.29	0.33	0.31	0.40	0.44	0.37	0.45	0.51	0
3-Ethyl-1,4-hexadiene	0.59	0	0	0	0	0	0.10	0.01	0	0
Benzaldehyde	0.90	0.21	0.27	0.16	0.06	0.16	0.19	0.21	0.20	0.04
1,3-Cyclooctadiene	0.32	0.16	0.14	0.11	0.11	0.15	0.22	0.15	0.12	0
(E)-Methyl 9-octadecenoate	0.22	0.22	0	0.07	0.04	0	0	0	0	0
Z-7-Hexadecylenic acid	0.35	0.06	0.08	0.09	0	0	0	0	0	0
Oleic acid	2.14	1.65	1.28	1.32	0.76	0.08	1.13	0.38	0.52	0
5-Methylundecane	0.77	0.23	0.02	0.42	0.46	0.26	0.53	0.16	0.43	0.16
**3-methyl-1-butanol**	0	0	0	0	0	0	0	0	0.24	0.53
3-Hydroxy-2-butanone	0.01	0	0	0	0	0.01	0	0	0	0.06
**1, 3-di-tert-butylbenzene**	0.08	0.49	0.57	1.01	1.06	1.74	0	1.91	1.98	1.98
**Acetic acid**	0	0	0	0	0	0	0.40	0.51	0.72	0.81
Pentadecane	0	0.10	0.17	0.29	0.35	0.35	0.22	0.27	0.31	0.21
2,3-Butanediol	0	0	0	0	0	0	0	0	0	0.32
Phenylethyl alcohol	0	0	0	0	0	0	0	0	0.01	0.13
3-methyl butyraldehyde	0	0	0	0	0	0	0	0	0	0.13

Table S3. Relative content changes of 31 volatile compounds of salmon during storage at 25 °C.

**Compounds**	**Relative content（μg/g）**					
	**0 h**	**24 h**	**36 h**	**48 h**	**60 h**	**72 h**
Methylmercaptan	0	0	0	0.87	0.91	0.96
3-methyl butyraldehyde	0.04	0	0	0	0.26	0.36
Dimethyl disulfide	0.02	0.02	0.03	2.21	1.26	4.56
1-Butanol, 3-methyl-, formate	0	0	0	0.48	0.22	0.26
Isovaleric acid	0	0	0.03	0	0.18	0.60
Phenylethyl alcohol	0	0	0	0.45	0.55	0.84
Phenol	0.07	0.02	0.03	0.48	0.47	1.01
Indole	0.03	0	0.01	0.22	0.06	1.74
**1, 3-di-tert-butylbenzene**	0.06	0.48	0.51	0.75	0.93	1.79
**Acetic acid**	0.04	0.04	0.11	0.65	1.18	1.57
Propanoic acid	0	0	0	0.08	0.13	0.11
Cyanoacetyl urea	0	0	0	0	0	0.07
2-butyl alcohol	0	0	0	0	0	0.12
Ethyl 2-methylbutyrate	0	0	0	0.06	0	0.38
n-propyl 3-methylbutyrate	0	0	0	0	0	0.09
**3-methyl-1-butanol**	0	0	0.09	1.15	0	1.63
Ethyl acetate	0	0	0	0	0	0.08
Phenylethylene	0	0	0.03	0.04	0.02	0.33
Dimethyl trisulfide	0	0	0	0.16	0.06	0.15
Nonaldehyde	0.15	0.13	0.11	0.06	0.05	0.03
Pentadecane	0.20	0.14	0.15	0.18	0.36	0.08
Heptadecane	0.15	0.11	0.14	0.15	0.10	0.09
α-pinene	0.14	0.17	0.12	0.17	0.08	0.01

Hexanal	0.99	0.93	0.61	0	0.86	0
3-carene	0.02	0.07	0.03	0.04	0.01	0
1-Penten-3-ol	1.19	0.12	0.09	0	0.07	0
1,2,3-trimethylbenzene	0.09	0.11	0.07	0.14	0.03	0
Octyl aldehyde	0.05	0.05	0.05	0	0.04	0
2,5-Octanedione	0.04	0.05	0.02	0	0	0
(E, E)-2,4-Heptadienal	0.26	0.29	0.27	0	0.19	0
Benzaldehyde	0.08	0.07	0.07	0.13	0.06	0.02

Table S4. The characteristic ions and retention times of 3-methyl-1-butanol, 1,3-di-tert-butylbenzene, Acetic acid under SIM mode.

Compound	Characteristic Ions（m/z）	RT（min）
3-methyl-1-butanol	55,70,41	8.144
1,3-di-tert-butylbenzene	175,190,57	14.339
Acetic acid	43,60	14.926
